# Establishing the safety of selective digestive decontamination within the ICU population: a bridge too far?

**DOI:** 10.1186/s13063-023-07356-3

**Published:** 2023-05-17

**Authors:** James C. Hurley

**Affiliations:** 1 Melbourne Medical School, University of Melbourne, Melbourne, Australia; 2 Division of Internal Medicine, Grampians Health Services, Ballarat, VIC Australia

**Keywords:** Bacteremia, Antibiotic prophylaxis, Herd peril, Study design, Intensive care, Mechanical ventilation, Selective digestive decontamination

## Abstract

**Background:**

Infection prevention interventions within the intensive care unit (ICU) setting, whether studied within quality improvement projects or cluster randomized trials (CRT), are seen as low risk and grounded in an ethical imperative. Selective digestive decontamination (SDD) appears highly effective at preventing ICU infections within randomized concurrent control trials (RCCTs) prompting mega-CRTs with mortality as the primary endpoint.

**Findings:**

Surprisingly, the summary results of RCCTs versus CRTs differ strikingly, being respectively, a 15-percentage-point versus a zero-percentage-point ICU mortality difference between control versus SDD intervention groups. Multiple other discrepancies are equally puzzling and contrary to both prior expectations and the experience within population-based studies of infection prevention interventions using vaccines. Could spillover effects from SDD conflate the RCCT control group event rate differences and represent population harm? Evidence that SDD is fundamentally safe to concurrent non-recipients in ICU populations is absent. A postulated CRT to realize this, the SDD Herd Effects Estimation Trial (SHEET), would require > 100 ICUs to achieve sufficient statistical power to find a two-percentage-point mortality spillover effect. Moreover, as a potentially harmful population-based intervention, SHEET would pose novel and insurmountable ethical issues including who is the research subject; whether informed consent is required and from whom; whether there is equipoise; the benefit versus the risk; considerations of vulnerable groups; and who should be the gatekeeper?

**Conclusion:**

The basis for the mortality difference between control and intervention groups of SDD studies remains unclear. Several paradoxical results are consistent with a spillover effect that would conflate the inference of benefit originating from RCCTs. Moreover, this spillover effect would constitute to herd peril.

## Introduction

Patients admitted to the intensive care unit (ICU) are at high risk of acquiring various infections arising from either their own microbiome or the ICU microbiome. Infections such as pneumonia and bacteremia increase the mortality risk [1–4]. Those receiving mechanical ventilation (MV) are at the highest risk and are the ICU population most often targeted within prevention studies.

There is an ethical imperative to prevent ICU-acquired infections towards ensuring safe and optimal care [5]. There is a broad range of potential ICU infection prevention interventions [6–26]. However, the pathway to demonstrating their safety and effectiveness for populations differs to the pathway for demonstrating their safety and efficacy for individuals.

### Precepts towards establishing population versus individual safety and efficacy

In developing vaccines, as with therapeutic drugs, extensive preclinical testing is essential to establish a firm basis for safety before undertaking human use studies [27, 28]. Only then are clinical trials commenced which, for a new vaccine, might be an evaluation in a randomized concurrent control trial (RCCT) with the objective of establishing both its efficacy towards preventing the target infection and its safety among individuals. For RCCTs, there are clear ethical principles and ethics review board approval together with informed consent from either participants or surrogate decision makers is mandatory. The individuals within an RCCT participate on an altruistic basis as they generally derive no benefit from the intervention.

Once vaccine safety is established for individuals, the cluster randomized trial (CRT), which is a well-established research tool, [29] is used to study the effectiveness and safety of the vaccine within populations. There are major statistical, conceptual and logistical differences between CRTs versus RCCTs. A CRT requires substantially more patients to achieve comparable statistical power due to the non-independence of observations within any one cluster. Moreover, the ethical issues arising within CRTs of interventions applied to populations differ across several domains from those raised by RCCTs of interventions applied to individuals [30–35].

In developing interventions to prevent ICU infections, there are some similarities but several points of difference to vaccine development. There is usually little role for preclinical testing, especially for interventions other than a drug, such as a checklist. For drug-based infection prevention interventions, the safety of the drug for individuals may have been established by its widespread therapeutic use over decades.

Some ICU infection prevention interventions might be studied within RCCTs, or if low risk, such as where the intervention is a checklist, within a quality improvement project (QIP) [36–43]. Individual participants within a QIP generally derive an indirect benefit in that the process contributes to ensuring that the care provided by the ICU is optimal and informed consent is usually waived [32, 41–43]. Moreover, QIPs generally report aggregate infection occurrences measured as incidence densities per unit time at risk rather than patient-level data.

Recently, several anti-microbial-based infection prevention interventions have been evaluated against ICU-acquired infections. Following evaluations of these interventions in multiple RCCTs, a few mega-CRTs have emerged with patient mortality as the primary endpoint and infection counts as secondary endpoints [44–47]. These mega-CRTs cluster randomized many (between 12 and 20) ICUs such that eligible patients in the intervention ICU receive the study intervention whereas eligible patients in the control ICUs might variously receive a placebo, an alternate infection prevention intervention, or standard care.

Here, several paradoxical discrepancies within the broader evidence base for antimicrobial-based ICU infection prevention interventions are described. These puzzling discrepancies, especially with respect to mortality, highlight that the population safety of selective digestive decontamination (SDD) remains undefined [48–52]. A postulated CRT to demonstrate the safety of anti-microbials used for infection prevention among ICU populations is described and the unique ethical and logistical issues that such a postulated trial would raise are considered.

Firstly, a clarification is required. Infection prevention among ICU patients receiving MV using topical applications of antibiotics as prophylaxis (TAP) is termed ‘selective digestive decontamination’ (SDD), along with ‘selective oropharyngeal decontamination’ (SOD) [53]. Confusingly, the term ‘selective digestive decontamination’ also describes the presumed mode of TAP action. Some regard the term ‘SDD’ as a triple misnomer and ‘control of gut overgrowth’ (COGO) is thought to better describe the underlying mechanism [54, 55]. Here, and elsewhere, [6, 7] topical antibiotic prophylaxis (TAP) is used to refer to the intervention as distinct from SDD, which is used to refer to the theoretical underlying concept.

### Discrepancies in the ICU infection prevention evidence base

The results of RCCTs of TAP are seemingly clear with significant summary effect sizes indicating apparent prevention of pneumonia, bacteraemia or mortality and other endpoints across over 40 RCCTs rated as good quality by the usual metrics of study design and blinding [6, 7, 56]. Of note, the possibility of a spill-over effect, wherein the intervention changes the risk of the event of interest occurring in the concurrent control group, is not considered among the parameters of RCCT quality. Here, it is the discrepancies in the mortality endpoint that is of particular interest although corresponding discrepancies in several microbiologically documented pneumonia and bacteraemia endpoints have been detailed previously [57–68].

These discrepancies are apparent only when these mortality and other event rates are compared to event rates expected for comparable ICU populations receiving MV. Groups within observational studies without an infection prevention intervention under study provide literature-derived external benchmarks for each event rate.

**Fig. 1:**
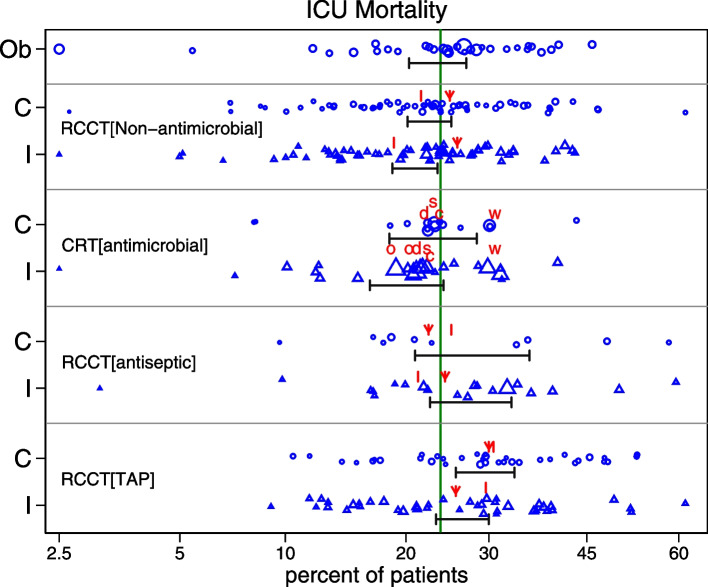
The ICU mortality incidence for the component (C = control ○; I = intervention Δ) groups (graded by group size) of individual studies of infection prevention interventions among patients receiving MV. Groups originating from RCCTs of interventions studied included non-antimicrobial-based methods (*n* = 81), topical anti-septic-based methods (*n* = 22) or topical antibiotic-based methods (*n* = 64). The overall benchmark being the summary mean (central vertical line) derived from the observational studies (Ob = observational; *n* = 43) is displayed together with the 95% confidence limits (CIs, horizontal error bars) associated with the summary incidence for each category. These 95% CIs were calculated using random effect methods as described in [52]. One control and one intervention group from each of five mega-CRTs (more than 10 ICUs) (c = CHORAL [69]; d = de Smet [44]; o = Oosterdijk [45]; s = SuDDICU [47]; w = Wittekamp [46]) and median control and intervention group mortality for five large Systematic reviews (more than 4 studies) (|= Bo [15]; ↓ = Gillies [12]; |= Zhao (Toothbrushing ± antispetic) [17]; ↓ = Hua (Topical Chlorhexidine) [16]; ↓ = Minozzi (TAP + PPAP) [7]; |= Minozzi (TAP alone)) [7] are indicated. Note the *x*-axis is a logit scale. The figure is adapted from reference [52]. and used here under the terms of the Creative Commons Attribution 4.0 International License (http://creativecommons.org/licenses/by/4.0/)

Table 1 lists four paradoxical observations that are evident within the median event rates as summarized within systematic reviews of ICU infection prevention interventions applicable to the MV patient group. Figure 1 illustrates the ICU mortality proportions from the individual studies abstracted within these systematic reviews together with the median estimates versus a literature-derived benchmark as derived in ref [52]. Of note, these four observations are paradoxical because, surprisingly, they are not inconsistent with the apparent prevention effect of TAP implied by the effect size estimates of any one of the listed TAP RCCTs or even the summary estimate of any one TAP systematic review. However, this inference is possible only when these estimates are examined in isolation and without scrutiny of the event rates underlying these estimates in relation to any literature-derived benchmarks [48–52, 57–68].

**Table 1:** Four paradoxical observations among systematic review summaries of various VAP prevention interventions

Intervention	Ref	VAP incidence (per 1000 patients)		RR; 95% CI	*n*/*N*	Mortality incidence (per 1000 patients)		RR; 95% CI	*n*/*N*	Figure 1 symbol
		Control	Intervention			Control	Intervention			
**Non-antimicrobial—RCCT**										
** Semi-recumbent** ^a^	[13]	316*	139^§^	0.44; 0.11–1.77	3/419	276*	240^§^	0.87; 0.59–1.27	2/307	
** HME** ^b^	[12]	167*	155^§^	0.93; 0.73–1.19	13/2251	247*	257^§^	1.03; 0.89–1.2	12/1951	↓
** Probiotic** ^c^	[13]	309*	238^§^	0.7; 0.52–0.95	8/1018	214*	186^§^	0.84; 0.58–1.22	5/703	׀
** Protocolized MV wean** ^d^	[23]	NR	NR	NR		216*	166^§^	0.77; 0.39–1.5	2/513	
**Antimicrobial – CRT**										
** TAP ± PPAP**	[56]	NR	NR	NR		See Fig. 1		1.00; 0.79–1.23^‡^	3/18335	
**Antiseptic – RCCT**										
** Chlorhexidine** ^e^	[16]	243*	180^§^	0.75; 0.62–0.91	18/2451	222*	242^§†^	1.09; 0.96–1.23	14/2014	↓
** Tooth brushing ± antiseptic** ^f^	[17]	259*	179^§^	0.61; 0.41–0.91	5/910	250*	210^§^	0.84; 0.67–1.05	5/910	׀
**TAP ± PPAP – RCCT**										
** TAP ± PPAP**	[56]	NR	NR	NR		NR	NR	0.85; 0.77–0.94^‡^	27/5699	
** TAP + PPAP** ^g,h,i^	[6]	NR	NR	0.28; 0.2–0.38	16/3024	NR	NR	0.75; 0.65–0.87^‡^	17/4075	
** TAP + PPAP** ^g,i,j^	[7]	417*	179^§^	0.43; 0.35–0.53	17/2951	303*	255^§†^	0.84; 0.73–0.96^‡^	18/5290	↓
** TAP (alone)** ^h,i,k^	[6]	NR	NR	0.34; 0.21–0.55	12/1735	NR	NR	0.97; 0.87–1.07	13/1783	
** TAP (alone)** ^i,j,k^	[7]	324*	162^§^	0.50; 0.36–0.69	13/1848	305*	296^§†^	0.97; 0.87–1.07	15/3274	׀

*VAP* Ventilator-associated pneumonia, *MV* Mechanical ventilation, *RR* Risk ratio, *95% CI* 95% Confidence interval, *n/N* Number of participants/number of studies, *NR* Not reported, *HME* Heat and moisture exchanger, *RCCT* Randomized concurrent controlled trial, *CRT* Cluster randomized trial, *MV* Mechanical ventilation, *TAP* Topical antibiotic prophylaxis, *PPAP* Protocolized parenteral antibiotic prophylaxis

^*^Paradox 1: median event rates for VAP and mortality among control groups of TAP (± PPAP)-RCCTs are generally high versus control groups of non-antimicrobial-RCCTs

^§^Paradox 2: median event rates for VAP and mortality among intervention groups of TAP (± PPAP)-RCCTs are not unusually low versus either intervention groups of non-antimicrobial-RCCTs or versus intervention groups of anti-septic RCCTs

^†^Paradox 3: median mortality event rates among chlorhexidine intervention groups are no higher than among TAP (± PPAP) intervention groups

^‡^Paradox 4: the mortality effect size for TAP (± PPAP)-RCCTs differs for studies with patients individually randomized versus cluster randomized, being equivalent to, respectively, a 15-percentage-point versus a zero-percentage-point difference between mortality in control and intervention groups

^a^Semi-recumbent position (30° to 60° versus < 10°); Pneumonia is microbiologically confirmed VAP at > 48 h and mortality is ICU mortality at > 48 h

^b^HME versus heated humidification: pneumonia measured at a median of 4 days (from Analysis 1.3 on page 65 of ref [12]) and mortality measured at a median of 8 days

^c^Probiotic versus control: pneumonia is VAP measured at a median 37 days and mortality measured at a median of 35 days

^d^Protocol-directed sedation management versus usual care for MV weaning

^e^Chlorhexidine (mouth rinse or gel) versus usual care: pneumonia is VAP measured at a median of 1 month and mortality measured at a median of 1 month

^f^Toothbrushing ± antiseptic: pneumonia is VAP measured at a mean of 1 month and mortality measured at a mean of 1 month

^g^TAP + PPAP intervention is equivalent to SDD (selective digestive decontamination). Summary results for seven studies (1039 patients) with a duplex design (TAP + PPAP versus PPAP) were not shown

^h^Data is based on analysis of intention to treat data including outcomes for patients lost to follow-up through early mortality for eight studies

^i^Pneumonia is a respiratory tract infection and the mortality is at unspecified follow-up

^j^Data is based on the analysis of per-protocol data

^k^TAP intervention is equivalent to SOD (selective oropharyngeal decontamination)

There are additional paradoxes. The results of the TAP studies vary due to the broad range of TAP formulations evaluated. Possibly, the failure to observe benefits in the CRTs could have been due to the choice of the TAP regimen. Puzzlingly, despite this broad range of TAP formulations, there is generally less variability among event rates within the TAP intervention groups than among the control groups of these RCCTs across various endpoints [48–52].

That the median mortality among intervention groups from fourteen RCCTs that studied topical chlorhexidine to prevent ventilator-associated pneumonia (VAP) in this patient group is no higher than that among intervention groups studies of TAP is surprising given the concern that topical chlorhexidine might increase mortality [16, 25].

### Herd (population) effects of infection prevention

The pattern of the discrepancy between the results of CRT and RCCT studies of TAP is contrary with respect to both prior expectations relating specifically to the theoretical SDD concept and also to the broad experience relating to herd effects of infection prevention interventions generally [27].

Herd effects, which benefit non-recipient individuals concurrently within populations exposed to vaccine interventions, are of great interest towards population-based vaccination programs but cannot be estimated within single populations examined in isolation [27]. Preventing the target infection among the vaccinated reduces the exposure and the infection risk for the concurrent control group members within any vaccine RCCT as a spillover effect [70, 71]. As a result, the vaccine effectiveness for the population will be underestimated.

The estimation of population-level effects of infection prevention interventions typically requires mega-CRTs with thousands of participants within dozens of exposed and unexposed neighbourhoods to achieve sufficient study power (Table 2) [44–47, 69, 72–74]. For example, CRT demonstration of typhoid vaccination herd effects required 60,000 residents in 40 neighbourhoods of Eastern Kolkata [72]. Sometimes several RCCTs can be stratified post hoc to simulate a single CRT. Demonstration of cholera vaccination herd effects was possible after post hoc re-analysis of an RCCT of 75,000 residents across five strata of neighbourhoods with incremental vaccine coverage with oral cholera vaccine (OCV) to enable simulation of a CRT [75].

**Table 2:** Published studies  depicted in Fig. 1 and the postulated SHEET described in the text

Author, year	Figure 1 symbol	Patients (*n*)/ICUs (*N*)^a^	Time periods Mo, months; w, weeks	Intervention	Mortality effect size^b,c,d^	ICC	Informed consent
Community CRTs							
Sur, 2009 [72]		60,000/40	24 mo	Typhoid vs hepatitis vaccination	NR	NR	Fever clinic attendees
Ali, 2005 [75]		74,003/[5 strata]	12 mo	OCV vs placebo vaccine	NR	NR	Post hoc re-analysis of RCCT data
MORDOR, 2018 [73, 74]		190,238/1533	24 mo	Azithromycin vs placebo	IRR 0.82; 0.74–0.90	NR	Oral or written
ICU CRTs							
De Smet, 2009^c ^ [44]	d	5939/13	6 mo × 3 crossover	SDD vs SC	OR; 0.91; 0.79–1.06 Adj OR; 0.81; 0.64–0.94	0.05	Waived
Oostdijk, 2014^c^ [45]	o	11,997/16	12 mo × 2 crossover	SDD vs SOD	OR; 0.94; 0.82–1.05 Adj OR; 0.87; 0.71–0.99	0.05	Waived
Wittekamp, 2018^d^ [46]	w	8665/13	6 mo × 4 crossover	SDD vs SC	Adj HR; 0.95; 0.81–1.11	0.001	Waived
CHORAL, 2021^f^ [69]	c	3260/6	2 mo × 6 steps; Step wedge	SC vs CHLX	OR; 1.13; 0.82–1.54	0.001	Waived
SuDDICU, 2022^ g^ [47]	s	5982/19	12 mo × 2 crossover	SDD vs SC	OR; 0.92; 0.79–1.08	0.01	Waived
SHEET (as postulated)^h^		15,600/104	12 mo × 2 crossover	SDD vs SC	TBD	0.001	TBD

*CHLX* Topical chlorhexidine, *SC* Standard care, *SDD* Selective digestive decontamination, *NR* Not reported, *NA* Not applicable, *RR* Risk ratio, *IRR* Incidence rate ratio, *OR* Odds ratio, *RD* Risk difference, *Adj* Adjusted, *TAP* Topical antibiotic prophylaxis, *PPAP* Protocolized parenteral antibiotic prophylaxis, *AS* Antiseptic, *OCV* Oral cholera vaccine, *TBD* To be determined

^a^These numbers (‘*n*’ = number of patients, and ‘*N*’ = number of clusters or studies) are for the whole study or systematic review, which in some cases include arms which are not shown here

^b^Mortality end-point is ICU mortality in all cases except MORDOR [73, 74] which is annual mortality. The study by Sur did not report mortality

^c^Adjusted analyses. 28-day mortality effect sizes from a random-effects logistic-regression model adjusted for age, sex, illness severity score and several other variables

^d^Adjusted analyses. The hazard ratio was estimated in analyses adjusted for age, sex, illness severity score and several other variables

^e^The following study arms are not shown here; de Smet study [44] also included SOD (Selective oropharyngeal decontamination) as a third arm versus standard care and the Wittekamp study [46] included SOD intervention as a fourth arm versus chlorhexidine bodywashings and a hand hygiene improvement program as the standard care arm

^f^The CHORAL study [69] evaluated the de-adoption of topical chlorhexidine. Note that the control group received topical chlorhexidine and the intervention group did not

^g^The ecological study within the SuDDICU study [47] is not shown here

^h^The RCCT within the postulated SHEET study is not shown here

Estimating herd effects in association with population-based vaccination interventions is facilitated if the infections are clinically distinctive, such as typhoid and cholera. This is not the case for ICU-acquired infections.

### Herd effects in the ICU setting

There are several reasons for expecting population-level effects from infection prevention interventions in the ICU setting.

Firstly, the expectation is inherent within the underlying postulates within the theoretical SDD concept as stated by Stoutenbeek in the first study in the ICU setting [61, 76]. Experiments in mice implicated a microbiome component that could be ‘selectively’ enhanced by exposure to specific antimicrobials and would contribute to colonization resistance within the digestive tract [77, 78]. Based on these experimental observations, this microbiome component, which remains unidentified, was expected to be transmissible between patients of an RCCT. Moreover, there was an expectation that the concurrent control group patients would recontaminate the decontaminated TAP-exposed intervention group patients to create an inverse spillover effect in the opposite direction.

Second, patients acquire colonization from contaminated surfaces within the hospital environment. For example, admission to a room previously occupied by a patient doubles the risk for acquiring pathogenic organisms from the previous patient’s flora [79–81]. Patients receiving TAP can serve as reservoirs for Pseudomonas and other Gram-negative bacteria within the ICU [82–87].

Herd effects of TAP are also evident with the rebound effect on withdrawal of TAP. The risk of severe infection on premature withdrawal of TAP had been noted in haematology units in the 1970s [88]. TAP had been used in the 1970s to prevent infections in association with neutropenia from cytotoxic chemotherapy. Rebound infections, which were often fatal, were observed in patients who had prematurely discontinued TAP due to its intolerable taste. More recently, rebound infection on TAP discontinuation has been noted among patients after ICU discharge when the infection risk is increased by some 50% [89].

Rebound may occur as a ‘whole of ICU’ phenomenon as it is not limited to the TAP recipients. Moreover, rebound might persist for several months after TAP withdrawal [83]. Patients remaining in the ICU after cessation of their TAP intervention may contribute to rebound in the ICU environment. Rebound underlies the importance of sufficient washout between consecutive periods of cluster randomized studies.

Could herd effects associated with TAP use within the ICU context explain the paradoxical findings and the discrepant results between RCCTs versus CRTs of TAP? To date, five studies have attempted to estimate the ecological effects of TAP in purpose-designed studies. Four were underpowered and generated inconclusive findings [90–93].

The fifth, the SDD in Intensive Care (SuDDICU) study, [47] studied three microbiological endpoints among non-recipients co-located alongside TAP or standard care recipients participating in a CRT to estimate the effect of TAP on mortality within 19 ICUs. No difference was found with respect to new positive blood cultures, new infections with antibiotic-resistant organisms or new *Clostridium difficile* infections between periods with TAP use versus periods with standard care among the co-located patients. There are four limitations with this ecologic assessment. The TAP regimen may have been ineffective as no significant effect was demonstrated on mortality in the CRT. Second, the eligibility for inclusion of co-located patients in the ecologic assessment was based mainly on non-eligibility for inclusion in the CRT of TAP versus standard care. Hence, the overall mortality risk of the co-located ecologic patients was half that of enrolled patients and their length of stay was not stated. Third, the ecologic endpoints were assessed in periods during the use of TAP in the ICU and immediately following its discontinuation. Endpoints occurring in the inter-period washout would have been subject to rebound on TAP discontinuation. Finally, mortality was not assessed among the ecological group.

### The ‘SDD Herd Effects Estimation Trial’ (SHEET) study

Given the pattern of discrepancies and the paradoxically high mortality within the control groups of RCCTs of TAP, it remains possible that any spillover effects could be harmful, potentially conflating the apparent benefit observed within RCCTs. Hence, it is crucial to clarify the safety of TAP use within ICU populations.

**Fig. 2:**
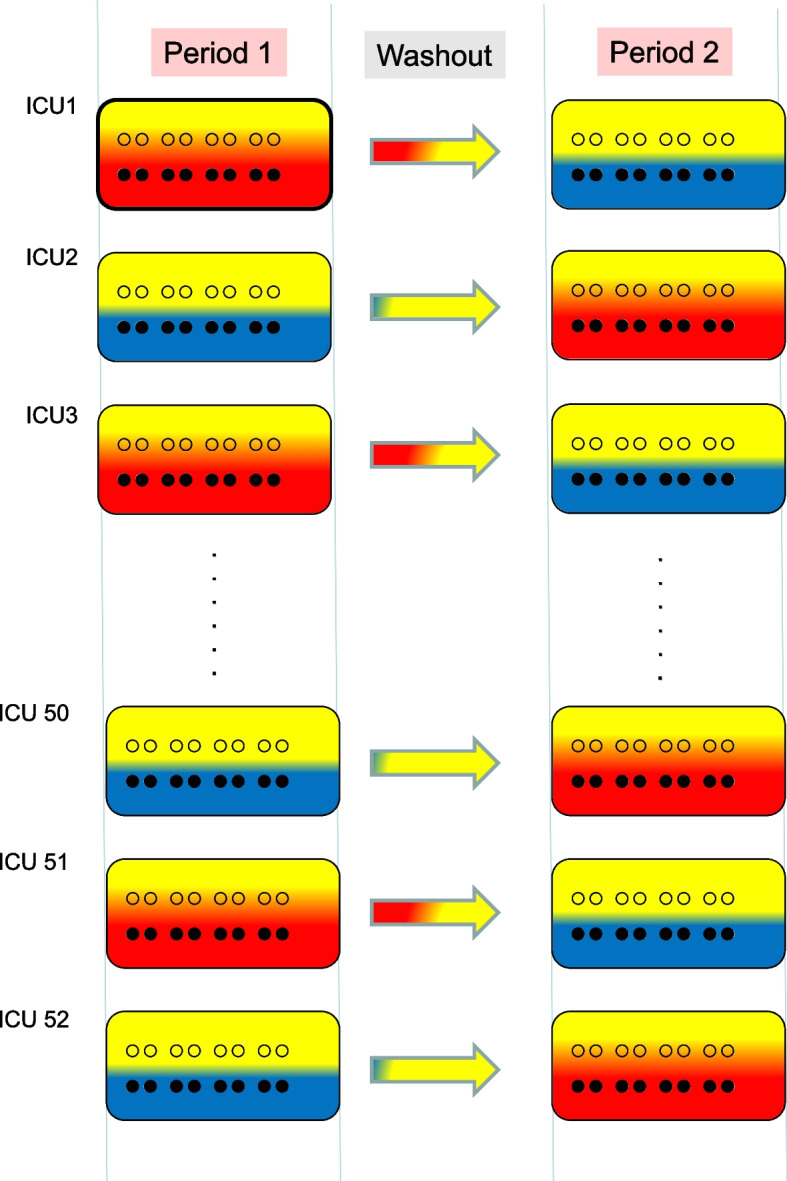
**SHEET trial design.** ICUs (*n* = 52) are cluster randomized to be either a TAP (half red ICUs) or a non-decontamination (i.e. non-antimicrobial) intervention (half blue ICUs) ICU in period 1 and to then cross over after a washout period. Within each ICU, all eligible MV patients are individually randomized to receive either the investigation agent (closed symbol ●) assigned to that ICU or not (open symbol ○). The SHEET trial is designed to enable the estimation of the indirect (population level) effect of TAP on mortality (spillover effect of the red colour onto the yellow) within ICUs. The washout period is necessary to enable any patients that have received the agent to be discharged from the ICU and any contextual effects associated with each investigational agent to dissipate. In previous CRTs, this period has been 3 months. Period 1 and period 2 are anticipated to be 12 months each. **SHEET trial analysis**. The RCCT component estimates the direct effect of each intervention at the level of individual patients by comparing the mortality among the patients randomly assigned to receive it or not within each ICU. The CRT component estimates the indirect effect of TAP at the population level by comparing the mortality among the patients randomly assigned to not receive the investigation agent (yellow half) within each ICU. The analysis presumes that the non-decontamination intervention will have no effect (direct or indirect) on mortality, as generally observed previously. The expected effects of TAP on mortality would be a lower mortality in those patient populations receiving TAP as the investigational agent versus those populations receiving the non-decontamination intervention. There is no capacity within the SHEET trial to estimate the possibility of any reverse spillover effect, that is the indirect effect from patients not receiving TAP on mortality (spillover effect of the yellow colour onto the red) within ICUs. This would require benchmarking the mortality among patients receiving TAP within the SHEET trial against the mortality among the four TAP CRTs in the literature

Spillover effects, being a contextual rather than an individual-level effect, can only be estimated within a purpose-designed CRT. This postulated CRT, hereafter referred to as the ‘SDD Herd Effects Estimation Trial’ (SHEET) study, is described. The logistical and ethical complexity of the SHEET study is such that it will likely not be undertaken (Fig. 2).

### SHEET: design considerations

The SHEET study would need to be simultaneously a CRT, with ICUs cluster randomized to be control or intervention ICUs, as well as an RCCT, with individual MV patients within each ICU randomly allocated to receive either direct or indirect exposures.

Within the intervention and control ICUs, the intervention would be either TAP versus an alternative method of VAP prevention, respectively. The control ICU intervention would ideally have a minimal ecological impact within the ICU, such as a non-antimicrobial-based intervention.

Within each control and intervention ICU, MV patients would be individually assigned by random allocation to receive the intervention assigned for that ICU or to receive standard care. The two layers of randomization within SHEET, which adds great logistical and ethical complexity, are required for two reasons.

First, the RCCT component enables estimations of the direct effects of the TAP and control interventions. It is essential that the TAP regimen chosen for study has activity comparable to that demonstrated within previous RCCTs.

Second, the CRT component enables an estimation of the ‘spillover’ effects from the assigned intervention to the concurrent patients in each ICU *not* receiving the assigned intervention. By comparing the mortality among those indirectly exposed to TAP within TAP ICUs versus that among those indirectly exposed within control ICUs enables estimation of the indirect effect of the TAP intervention. This is the central research question of the SHEET study.

### SHEET: statistical power calculations

A two-percentage-point mortality benefit, as reported from two previous CRTs of TAP [44, 45] (although this required an analysis adjusted for individual patient-level risk factors to achieve statistical significance) can be taken as a clinically significant difference that might be discoverable by a CRT whether as a mortality benefit, or harm, to non-recipients within the ICU as a spillover effect.

A group size of up to 200 (100 per group within each ICU) would be achievable. For example, the recruitment rate achieved within SuDDICU was approximately 150 patients per 12-month period per ICU [47] and the median group size in the prior RCCT-TAP experience was 80 patients (160 per study) [6, 7]. In regard to group size with SHEET, minimizing the number of patients exposed to potential harm requires careful consideration [94].

A crossover design is often used in CRTs to maximize the study power. This would require care as the ecological effects of TAP persist for possibly weeks or months. Previous CRTs of TAP had washout periods between one period to the next to ensure no carryover of contextual effects. A test for treatment by period interaction would be required to test for carryover of the spillover effect. Topical placebos to achieve study blinding would not be used as their population effect in the ICU context is uncertain [68].

**Fig. 3:**
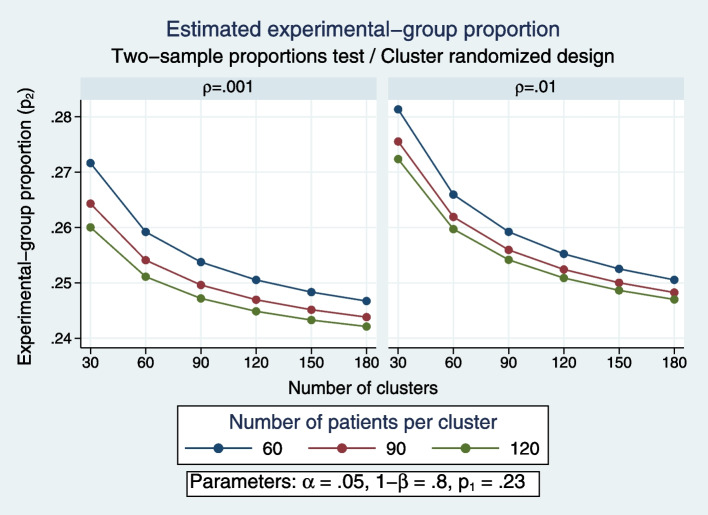
A benchmark ICU mortality of 23% as derived previously is used to represent the background mortality rate without intervention [52]. The intra-cluster coefficient (ICC) used in power calculations in previous mega-CRTs ranged between 0.001 and 0.01 (Table 2). Using these estimates, a study with 104 clusters (ICUs) each with 150 patients (75 directly and 75 indirectly exposed), (or 52 ICUs with a two-period crossover design), and an ICC of 0.001 (left panel), would have an 80% power to detect a 2% absolute increase (or decrease) in mortality at a 0.05 level of significance (using Stata command ‘power twoproportions .23 .25, m1(75) m2(75) rho(0.001)’). A two-period study with crossover would require 52 ICUs for the same power. With an ICC of 0.01 (right panel), the number of ICUs required increases to 168 (‘power twoproportions .23 .25, m1(75) m2(75) rho(0.01)’) (or 84 ICUs for a two-period crossover)

Hence, large numbers of both patients and ICUs will be required to achieve adequate study power with both patient and cluster randomization to ensure adequate control of the many potential confounders at the levels of both individual patients and ICUs (Fig. 3).

The design of the SHEET, as with all studies of infection prevention interventions in the ICU setting, is challenged by the following additional factors. Infections in the ICU setting, whether acquired from the patient’s microbiome or the ICU microbiome, might occur at any of several sites, such as bacteremia or pneumonia; might be caused by any of several microbes, such as bacteria like Pseudomonas or fungi such as Candida; and might be identified by different diagnostic criteria in different ICUs. Moreover, some criteria used in the diagnosis of pneumonia can be criticized as being inherently subjective. Observer blinding may be difficult or impossible to achieve.

Assessing the mortality endpoint is confounded by the heterogeneity in the underlying illness of individual patients in ICUs as well as heterogeneity in the admission and management policies of different ICUs. Adjustment for differences in underlying patient risk to increase the study power would be avoided as this adjustment would not account for the contextual risk, which can only be measured at the group level.

### Ethical precedents

There are four CRTs, two community-based [72, 73] and two ICU-based, [69, 95] that provide precedent to the SHEET study design (Table 2).

In the study of typhoid vaccination herd effects within the neighbourhoods of Kolkata, it was reasonable to expect, given the extensive vaccination knowledge base, [27] that any herd effects from typhoid vaccination could only be beneficial within the Kolkata neighbourhoods [72].

The MORDOR (Macrolides Oraux pour Réduire les Décès avec un Oeil sur la Résistance) study, a placebo-controlled CRT of azithromycin administration to prevent childhood mortality in Sub-Saharan Africa, examined over 320,000 person-years across over 1500 communities to demonstrate lower childhood mortality in association with the mass distribution of azithromycin [73]. A sub-study of complier average causal effect found a non-significant nine-percentage-point lower mortality among non-compliers as a spillover effect within the clusters randomized to receive azithromycin [74].

The Keystone Study is a prospective study involving 103 ICUs that ascertained changes in the incidence density of bloodstream infections resulting from central venous catheters in response to interventions known to reduce catheter-related infections in their patients [95]. The nature of the intervention in the Keystone study, a five-point checklist of CDC-endorsed practices, was directed at the ICU health professionals’ behaviours in their use of these procedures. Whilst the Keystone study results indicated the potential to halve the rates of catheter-related infections, there was no expectation that this intervention would engender herd effects. A requirement for informed consent from participants was waived by the IRB.

The CHORAL study was undertaken alongside a de-adoption of chlorhexidine among six Canadian ICUs [69]. This de-adoption was planned in response to emerging evidence of potential harm from topical chlorhexidine use. This planned de-adoption enabled a stepped-wedge design CRT, wherein sequential crossover of clusters from control to intervention occurs until all clusters are exposed to the intervention [96]. Here, the intervention was the non-use of topical chlorhexidine. Informed consent was not required as the de-adoption was a planned change in the standard of care.

With the SHEET study, there is an expectation of SDD herd effects but no basis for predicting whether these might be beneficial, harmful or neutral within the ICU setting. These uncertainties raise unique ethical considerations across at least six domains for the SHEET study [30–43, 97, 98].

### Who is the research subject? [30, 31]

A research subject could be defined as ‘an individual whose interests may be compromised as a result of interventions in a research study’. This exposure need not be direct. An indirect intervention that manipulates the environment, such as with a spillover effect, might equally compromise an individual’s interests.

By contrast, QIP participants are generally not considered research subjects even though their outcomes are being measured. In a QIP there is an intent to ensure that patient care in the ICU is optimal and the QIP is in the interest of all patients of the ICU, including the ones participating. Given the central research question within the SHEET study, all patients would likely be considered research subjects.

### From whom, how and when must informed consent be obtained? [30, 32]

The SHEET study will raise complex consent issues. Within the RCCT component of the SHEET study informed consent would be obtained, as has been the case in every RCCT of TAP undertaken to date.

For the CRT component, informed consent could only be waived if four conditions were present: (1) the research cannot ‘practicably be carried out’ without the waiver; (2) the subjects' rights and welfare will not be adversely affected; (3) the research involves no more than minimal risk; and (4) subjects will be provided with additional pertinent information after participating. The SHEET study would likely meet only the first of these conditions.

On the other hand, it is often impractical to obtain informed consent from all CRT participants. Whether informed consent from the participants in The Keystone study should have been obtained was widely discussed. However, the Keystone study can be seen as a mega-QIP of ICUs under different management strategies rather than a study of individual patients within those ICUs [95, 97]. The Keystone study has been replicated, and participant informed consent was again not required by the IRB [98].

For the study of typhoid vaccination, presentation with fever to health centres within each of the Kalkoota neighbourhoods was the primary endpoint. Oral consent was obtained from those who presented with fever [72]. For the SHEET study, obtaining informed consent from those who experience the primary endpoint (mortality) is clearly problematic.

For the SHEET study, in contrast with the above, a difference in patient mortality is anticipated. Addressing the primary research question of the study, being the direction of the mortality difference, whether an increase or a decrease, can only be ascertained from a mortality count derived from individual patients. In obtaining informed consent, what information should be conveyed and to who? There are risks and benefits of receiving TAP and there are also possible risks and benefits to bystanders not receiving TAP. If a potential participant declines to enter the SHEET study, what then? Presumably, they require removal from the study ICU, with relocation to an ICU not engaged in the study, to avoid indirect exposure within an ICU participating in the SHEET study.

### Does ethical equipoise exist? [30, 33]

The risks and benefits of SDD use are open to debate both for individual patients and for the ICU population.

On the one hand, SDD is an intervention which, based on the RCCT evidence, has arguable individual patient benefit and to not use it is thought by some to be unethical [99]. By contrast, there is a clear divide within both European and American critical care communities over whether decontamination-based infection preventions are appropriate, whether the risks outweigh any benefits, and which decontamination regimen might be used. For some jurisdictions, the concern regarding the potential to increase antibiotic resistance through antibiotic overuse is the overriding consideration and SDD is not used. For example, in the US, topical chlorhexidine is more commonly used as an infection-prevention intervention within ICUs [53, 100]. Even with chlorhexidine there is uncertainty about the risks versus benefits with some unexplained increased mortality occurring as a direct effect identified in a post hoc analysis in some ICU populations [25].

A survey of European ICUs found that SDD was used in only 35 (16%) of 237 ICUs. Even within the Netherlands, where SDD originated, only 13 of 23 ICUs reported the use of SDD [101]. Moreover, within this same survey, fewer than 20% of European intensivists were able to agree with a statement that there was evidence to support SDD use. European consensus guidelines suggest ‘..the use of SOD, but not SDD, in settings with low rates of antibiotic-resistant bacteria and low antibiotic consumption.’ This suggestion was provided as a weak recommendation on the basis of low-quality evidence [102]

### Are the risks and benefits balanced? [29]

For a non-drug intervention, such as a checklist as in the Keystone study, the risk to individuals and populations participating in the study should be zero. Arguably, the risks of not participating in such a study could be greater. The counterargument would be that on this basis, checklists could be introduced to standard practice without a formal study of their effect on infection incidence densities. Checklists have become standard practice to enhance safety within the aviation industry without a formal evaluation.

The safety of TAP as infection prevention for individual recipients cannot be established until the population safety of TAP use within the ICU is proven. The few preclinical studies that are available to be cited as a basis for the SHEET study do not provide clarity on the balance between risks versus benefits. Potentially, exposure to TAP changes the microbiome of the ICU and increases the risk for invasive infections as a result of colonization susceptibility [103–106].

SDD regimens require an anti-fungal component to avoid fungal overgrowth resulting from the TAP. Whether these anti-fungal components are sufficient to counter this fungal overgrowth remains unclear. From the perspective of the individual, the taste of SDD is poorly tolerated causing some patients to refuse to take it once they had regained the ability to do so.

It remains possible that the microbiome changes at the individual and population level resulting from TAP use may provide both individual benefits directly to the recipients of TAP as well as population harm occurring among those in the ICU not receiving it. This raises an ethical dilemma termed ‘Trolleyology’ [107]. An example of Trolleyology is the dilemma of population Dengue vaccination programs which may provide great population benefit but with a risk of known harm occurring to an unknown minority [108].

### Are there vulnerable groups to be protected? [30, 34]

ICU populations are inherently vulnerable because of the underlying illness causing their ICU admission. Moreover, the patient population receiving MV is of great research interest but is also the most vulnerable and, given they are intubated and ventilated, unable to engage in conversations of the risks and benefits of study participation.

For studies undertaken within ICU populations, informed consent is often sought from surrogate decision makers such as the patient’s next of kin where the patient is unable to give informed consent.

### Who are the appropriate gatekeepers? [30, 35]

Who should be the ultimate guardians or gatekeepers who determine whether the SHEET proposal is acceptable to proceed?

Single-centre infection prevention interventions undertaken as a QIP are generally deemed as low risk and acceptable to proceed by IRBs. For multi-site QIPs, a single ethics review committee might review a proposed study protocol and decide on behalf of multiple study sites as with the Keystone CRT. Some have argued that multi-centre CRTs of infection prevention interventions undertaken across more than one country have the same imperative as a QIP and should not be unnecessarily impeded by regulatory obstacles, such as an IRB [109].

Others might argue for greater oversight. In the case of the typhoid vaccination and the MORDOR CRTs, oversight was provided independently by both local and international ethics review committees in each case [72, 73].  For the SHEET trial, it is likely that ICU recruitment would need to be across more than one country to obtain the required numbers.

In the case of the SHEET study, the questions are more complex given the central research question relates to mortality as the primary endpoint. The gatekeepers would be keen to ‘first do no harm’, especially so for an intervention that is merely preventive rather than therapeutic. Finally, the duty of care owed by hospitals to their patient populations is a duty that could not be delegated.

## Discussion

The protocol for the postulated SHEET study illustrates the unique logistical and ethical challenges that would arise in establishing the population safety of an antimicrobial-based infection prevention intervention, like TAP, within the ICU context. There is much speculation regarding the population effects of TAP but no evidence to refute the concern, arising from multiple paradoxical observations, of harmful spillover effects from TAP use in the ICU context.

Unlike therapeutic interventions, the individuals who derive benefit or harm from population-based preventive interventions, such as TAP use, whether as a direct or indirect effect, are not readily identifiable. Overriding the imperative to develop better infection prevention methods for patients at risk in the ICU is the greater ethical imperative to first do no harm.

An alternative to the SHEET study is a de-adoption study as in the CHORAL study. The de-adoption incurs the risk that any infection rebound on the withdrawal of TAP from routine use would distort the assessment. Another approach would be to combine the RCCTs undertaken to date to simulate a single CRT, as was done in the case of evaluating the population effect of the oral cholera vaccine (OCV) among RCCTs with different levels of coverage [75].

## Conclusion

In the evaluation of ICU infection prevention interventions, evidence of safety and efficacy for individuals is only the first step. Demonstrating safety and effectiveness for populations is an essential second step and this requires a CRT. The postulated SHEET study, a hypothetical CRT to evaluate the safety and confirm the efficacy of TAP interventions in the ICU setting, will require thought as to who the research subjects are, whether informed consent is required and from whom, whether there is equipoise, the benefit versus the risk, considerations of vulnerable groups and who is to be the gatekeeper. The SHEET study is unlikely to be undertaken leaving the population safety of TAP unresolved and its efficacy unclear.

### Panel: Search strategy

I searched the Cochrane Library from Jan 1, 2012, to Dec 7, 2022, for systematic reviews of infection prevention interventions applicable to patients at risk of acquiring infections whilst receiving prolonged mechanical ventilation whilst in the intensive care unit. I used search terms related to the prevention of infection, whether antimicrobial-based or non-antimicrobial-based methods and whether limited to the patient population receiving MV or expanded to the population at risk of receiving MV such that > 50% of the included population received MV. Systematic reviews limited to interventions applicable to specialized populations, such as paediatric or patients with Adult respiratory distress syndrome (ARDS), were excluded. Since systematic reviews generally include only RCCTs the search was supplemented by a search for CRTs of methods of ICU infection prevention. CRTs and additional systematic reviews and meta-analyses were identified using the literature as cited by the most recent CRT together with a search using the related article function in Google Scholar.

This search yielded 14 systematic reviews and meta-analyses [6–18, 23] including several published since earlier literature searches [57–59]. Large systematic reviews (> 4 studies) that reported ICU mortality and mega-CRTs (> 10 ICUs) that reported ICU mortality were of specific interest in the quantitative synthesis and benchmarking of ICU mortality for the MV patient group in the literature (as presented in Fig. 1) [6–26, 44–47, 69]. I also reviewed the results from literature searches for related topics [110, 111, 112].

## Abbreviations

CRT: Cluster randomized trial
ICU: Intensive care unit
Keystone Study: Michigan Health and Hospital Association Keystone Intensive Care Unit Study
RCCT: Randomized controlled trial
COGO: Control of gut overgrowth
MV: Mechanical ventilation
OCV: Oral cholera vaccine
PPAP: Protocolized parenteral antibiotic prophylaxis
NCC: Non-concurrent control
CC: Concurrent control
SOD/SDD: Selective oropharyngeal decontamination/selective digestive decontamination
SHEET: SDD Herd Effects Estimation Trial
TAP: Topical antibiotic prophylaxis
